# Bridging postural biomechanics and knee arthroplasty success

**DOI:** 10.1186/s42836-026-00411-9

**Published:** 2026-07-24

**Authors:** Filippo Migliorini, Nicola Maffulli, Luise Schäfer, Philipp Kobbe, Andreas Bell, Raju Vaishya, Jörg Eschweiler

**Affiliations:** 1 Department of Trauma and Reconstructive Surgery, University Hospital of Halle, Luther University Halle-Wittenberg, 06120 MartinHalle (Saale), Germany; 2 Department of Life Sciences, Health, and Health Professions, Link Campus University, Via del Casale Di San Pio V, 00165 Rome, Italy; 3 Department of Orthopaedic and Trauma Surgery, Eifelklinik St. Brigida, Kammerbruchstr. 8, 52152 Simmerath, Germany; 4 Department of Trauma and Orthopaedic Surgery, Faculty of Medicine and Psychology, University “La Sapienza” of Rome, 00189 Rome, Italy; 5 School of Pharmacy and Bioengineering, Faculty of Medicine, Keele University, Stoke On Trent, ST4 7QB UK; 6 Centre for Sports and Exercise Medicine, Barts and the London School of Medicine and Dentistry, Queen Mary University of London, Mile End Hospital, 275 Bancroft Road, London, E1 4DG UK; 7 Department of Trauma and Reconstructive Surgery, BG Klinikum Bergmannstrost Halle, 06112 Halle (Saale), Germany; 8 Department of Orthopaedics and Joint Replacement Surgery, Indraprastha Apollo Hospital, Sarita Vihar, 110076 New Delhi, India

**Keywords:** Pelvic tilt, Replacement, Sagittal alignment, Spinopelvic mobility, Coronal imbalance, Biomechanics, Alignment

## Abstract

Despite advances in surgical techniques and implant design, a significant number of patients remain dissatisfied after total knee arthroplasty (TKA), often because of factors unrelated to the arthroplasty itself. Among these, pelvic biomechanics play a vital yet frequently overlooked role in affecting knee alignment, load distribution, and functional outcomes. This review explores how pelvic orientation, tilt, and dynamics, as well as coronal asymmetries and spinopelvic mobility, influence knee biomechanics and TKA planning. By viewing the pelvis not as a static reference but as a dynamic biomechanical driver, this study discusses how sagittal imbalance, pelvic obliquity, and stiffness impact intraoperative assessment and postoperative knee function. Using an integrated, evidence-based approach, the present study advocates for a paradigm shift in knee arthroplasty that recognises the pelvis as a central factor for success in TKA and promotes personalised solutions grounded in whole-body biomechanics.

## Introduction

Total knee arthroplasty (TKA) is a common procedure in orthopedics [1–3]. Despite correct implant positioning and standardised optimal surgical technique, up to one in five patients remains dissatisfied. Traditionally, surgical planning for TKA has concentrated on geniculate mechanics [4–6], often overlooking the contributions of the pelvis and spine [7, 8]. Growing evidence suggests that pelvic biomechanics contribute to sagittal and coronal alignment, compensatory mechanisms, and soft-tissue tension [9–11]. The pelvis connects the spine and lower limbs both anatomically and functionally, and its orientation affects the mechanical axis, joint loading, and muscle activation along the kinetic chain [12–14]. Any deviation from optimal alignment requires compensatory adaptations throughout the lower limb, which often involve the knee joint [14, 15].

Pelvic tilt, frequently described in the sagittal plane, is a dynamic biomechanical parameter of clinical significance rather than a fixed postural label [16, 17]. Misinterpreting tilt as a static entity risks oversimplifying assessment and can result in suboptimal surgical planning [18]. When pelvic rotation surpasses normal adaptive limits, the hip–knee–ankle axis and the flexion–extension relationship may change, potentially affecting femoral component alignment, extensor mechanism function, and patellar tracking [19–21]. Beyond sagittal dynamics, coronal pelvic parameters also influence lower limb alignment and knee biomechanics [20]. Pelvic obliquity and coronal trunk asymmetry, whether structural or compensatory, can impact the apparent hip–knee–ankle axis, abduction–adduction moments at the knee, and the subjective perception of leg length [22, 23]. Mild lumbar-sacral scoliosis or asymmetric pelvic positioning can induce functional limb length discrepancy and coronal knee adaptations, which may be mistaken for primary joint deformities if assessed in isolation [23]. A detailed discussion of spinal deformity is beyond the scope of this review, but recognising coronal pelvic behaviour is vital for a comprehensive understanding of knee alignment within the overall kinetic chain.

This review discusses how sagittal and Coronal pelvic parameters and spinopelvic mobility influence TKA outcomes, with particular attention to distinguishing static descriptors from dynamic compensations and promoting a functional interpretation of pelvic alignment. For clarity, it is important to distinguish between commonly used alignment paradigms in TKA. Mechanical alignment aims to restore a neutral hip–knee–ankle axis, assuming symmetrical load distribution as an optimal target [24, 25]. Kinematic alignment aims to reproduce the patient’s native joint geometry by restoring pre-arthritic alignment and joint-line orientation. Functional alignment represents an evolution of these concepts, integrating bony anatomy with soft-tissue balance and global posture [26–28]. This philosophy allows controlled deviation from neutral alignment to accommodate individual biomechanical characteristics [29–32]. These paradigms differ in their underlying assumptions about normality, compensation, and adaptability and serve as the theoretical background for the present review. Much of the evidence linking pelvic biomechanics to knee outcomes derives from observational, modelling, or imaging-based studies; the mechanistic relationships discussed below should therefore be interpreted as biomechanical hypotheses rather than established causal links.

### Pelvic biomechanics and sagittal alignment

**Fig. 1:**
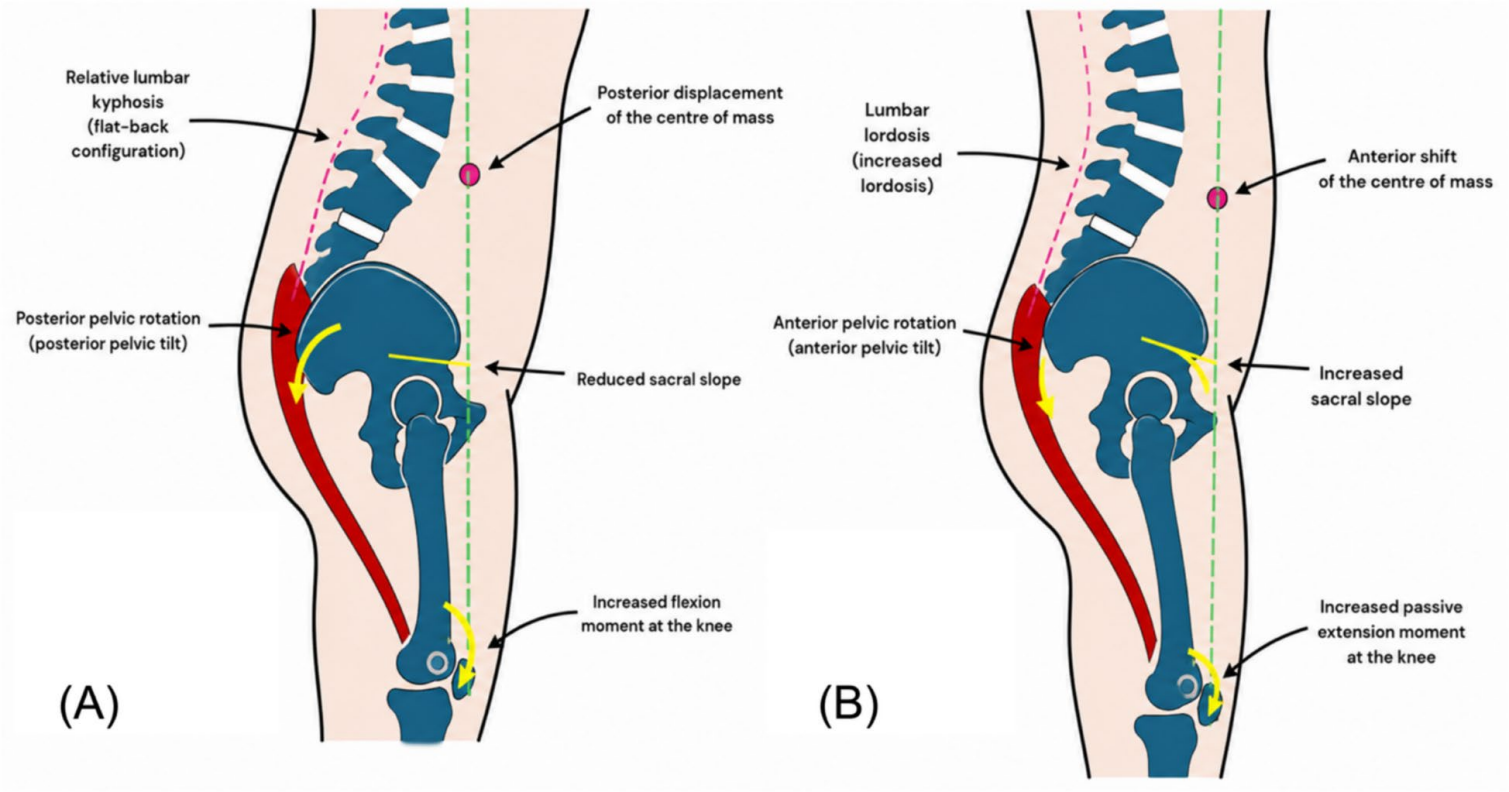
Pelvic tilt and sagittal compensation mechanisms. **A** Posterior pelvic tilt represents a compensatory adaptation to a sagittal spinal imbalance, characterised by posterior pelvic rotation, reduced sacral slope, relative lumbar kyphosis (flat-back configuration), posterior displacement of the centre of mass, and an increased flexion moment at the knee. Overall, pelvic orientation reflects spinal geometry within a hierarchical sagittal compensation strategy rather than acting as an independent primary driver. **B** Anterior pelvic tilt is characterised by anterior rotation of the pelvis with an increased sacral slope and lumbar lordosis, resulting in an anterior shift of the centre of mass and an increased passive extension moment at the knee

The pelvis acts as a biomechanical centre, transmitting forces between the spine and lower limbs while adjusting sagittal balance through ongoing postural adjustments [33, 34]. Its sagittal alignment, primarily controlled by pelvic tilt, directly influences spinal curvature, femoral orientation, and knee position [35, 36]. Tilt shifts dynamically during everyday activities and also varies between standing and sitting [36]. Figure 1 provides a schematic overview of sagittal pelvic compensation patterns and their potential influence on knee alignment.

**Fig. 2:**
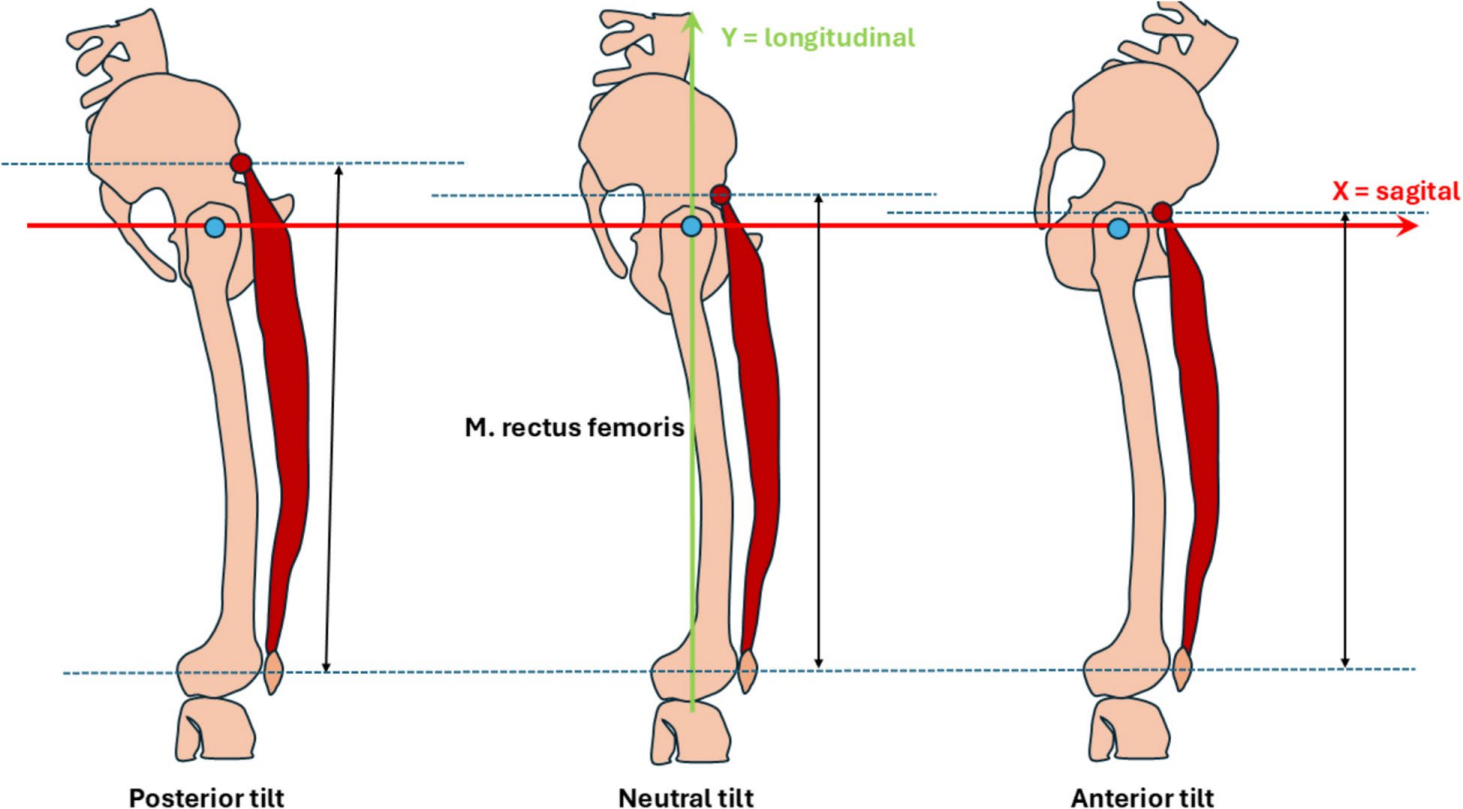
Effects of anterior and posterior pelvic tilt on sagittal posture, centre of mass, and knee mechanics

Anterior pelvic tilt increases lumbar lordosis and shifts the centre of mass (CoM) forward [37, 38]. Anterior pelvic tilt refers to the sagittal rotation of the pelvis in space (Fig. 1). It is best interpreted as a functional measure, typically quantified by pelvic tilt or sacral slope during weight-bearing [39]. While the anterior pelvic plane (defined by the ASIS and pubic symphysis) offers an anatomical reference, it does not reflect the dynamic postural behaviour relevant to knee biomechanics [40]. Anterior pelvic tilt should not be viewed as an inherently compensatory posture. Instead, it may indicate either a primary alignment pattern or a secondary adaptation, depending on the cause of the sagittal imbalance. In patients with primary lumbar hyperlordosis, anterior pelvic rotation often follows spinal alignment changes and can be managed at the hip level, provided adequate hip extension reserve remains [41]. Conversely, in patients with primary hip flexion contracture, increased lumbar lordosis and anterior pelvic tilt develop as compensatory mechanisms to sustain upright posture and horizontal gaze [41]. In this scenario, the hip’s limited ability to extend shifts the compensatory requirement downward, making femoral and knee flexion functionally necessary rather than optional [42]. Failure to identify hip flexion contracture may lead to misinterpreting knee deformity as primary, with significant implications for surgical planning in TKA. To decrease shear forces and improve joint congruity, the femur increases anteversion and external rotation by activating the quadriceps and ileopsoas, and the knees respond with slight extension [43]. This pattern is common in patients with disc degeneration or muscular imbalance [44]. It results in overuse of calf muscles, particularly the gastrocnemius and soleus, which can reduce ankle dorsiflexion [45]. At the knee, anterior tilt increases femoral internal rotation and dynamic valgus, promoting lateral patellar tracking and raising stress on the patellofemoral joint [46]. The rectus femoris muscle becomes functionally shortened, disrupting quadriceps balance and impairing patellar alignment, and has been associated with patellofemoral pain [46] (Fig. 2).

Anterior pelvic tilt shifts the CoM of the body forward, but the ground reaction force vector at the knee tends to move posteriorly relative to the joint [35]. This changes sagittal-plane mechanics, increasing the passive extension moment at the knee [35]. Consequently, there is greater tension on the posterior capsule and a chronic low-grade strain on the posterior cruciate ligament, which may shorten adaptively. This posterior force also influences femoral-tibial kinematics and patellofemoral tracking issues [35, 47].

On the opposite side, posterior pelvic tilt flattens the lumbar curve, “kyphosis”, shifts the centre of mass backwards, and may increase femoral retroversion and internal rotation, which may contribute to knee hyperextension [37, 38]. Posterior pelvic tilt should be interpreted predominantly as a compensatory adaptation rather than a primary postural pattern. In most clinical cases, a loss of lumbar lordosis, flat-back deformity, or thoracolumbar kyphosis occurs before and causes posterior pelvic rotation as part of a comprehensive sagittal balance strategy [44]. In this scenario, the pelvis retroverts to reposition the CoM and maintain an upright posture, which reduces the sacral slope [44]. Consistent with the principles outlined by Dorr and others, pelvic orientation reflects spinal geometry rather than functioning as an independent driver [48, 49]. The key biomechanical differences between anterior and posterior pelvic tilt, including their effects on spinal alignment, centre of mass, and knee loading, are summarised in Table 1.

**Table 1:** Main biomechanical considerations of the pelvic tilt

Feature	Anterior Pelvic Tilt	Posterior Pelvic Tilt
Lumbar spine	Increased lumbar lordosis	Reduced lumbar lordosis (relative kyphosis)
Centre of mass	Shifted anteriorly	Shifted posteriorly
Hip/femur	Increased femoral anteversion and hip flexion	Increased femoral retroversion and internal rotation
Knee posture	Slight flexion, dynamic valgus tendency	Hyperextension tendency
Ground reaction force at knee	Posterior to knee, increased passive extension moment	Anterior to knee, increased flexion moment
Capsuloligamentous strain	Increased posterior capsule and PCL strain	Increased anterior capsule and ACL stress
Muscle balance	Rectus femoris shortening, calf overuse	Increased quadriceps activation
Patellofemoral mechanics	Lateral tracking and increased PF stress	Increased PF contact pressure

**Fig. 3:**
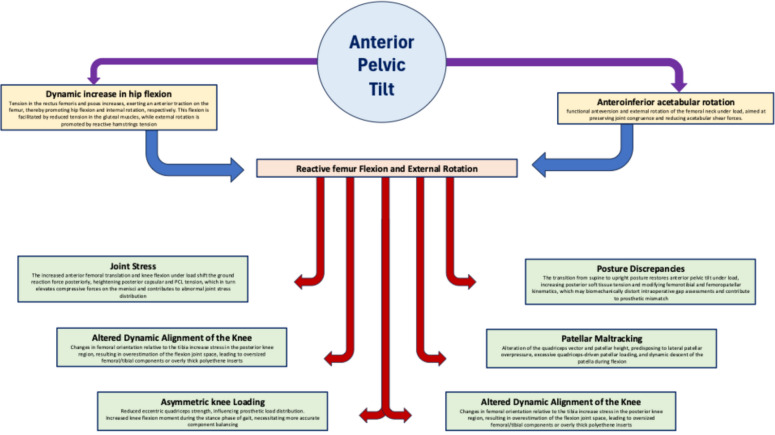
Biomechanical implications of anterior pelvic tilt

Failing to recognise this hierarchy may lead to misinterpretation of knee flexion posture as a primary deformity, thereby affecting alignment targets and intraoperative decisions during TKA. When these compensations become extreme, they can become maladaptive. This shift alters the ground reaction force vector anterior to the knee joint, creating a flexion moment that the body compensates for by increasing quadriceps activation and placing greater strain on the anterior joint structures [38]. The changed mechanics place increased stress on the anterior capsule and possibly on the anterior cruciate ligament (ACL) [50]. In the lower leg, compensatory strategies may involve excessive dorsiflexion and tibial internal rotation, disrupting normal load distribution and increasing strain on the anterior knee. Patellofemoral contact pressure may rise as a consequence of the altered patellar movement and higher compressive forces during flexion [51]. Patients with significant posterior tilt and thoracolumbar kyphosis often maintain persistent knee flexion to preserve horizontal gaze [52, 53]. If not recognised, surgeons might mistake this for fixed contracture, leading to unnecessary changes in distal femoral resection or posterior slope, and risking gap imbalance or joint-line elevation [54]. Conversely, excessive anterior tilt can tighten the extensor mechanism, drive the femur into relative flexion, and displace the patella, causing anterior pain or maltracking. These changes, which may be missed on standard knee radiographs, can be seen on full-body imaging [20, 55]. Spinopelvic stiffness, common after lumbar fusion or in ankylosing spondylitis, further limits compensation, shifting demands to the hips and knees, and increasing the risk of flexion contractures and early fatigue [56]. The knee-level biomechanical consequences of exaggerated anterior pelvic tilt, which are particularly prone to intraoperative misinterpretation, are illustrated in Fig. 3.

**Fig. 4:**
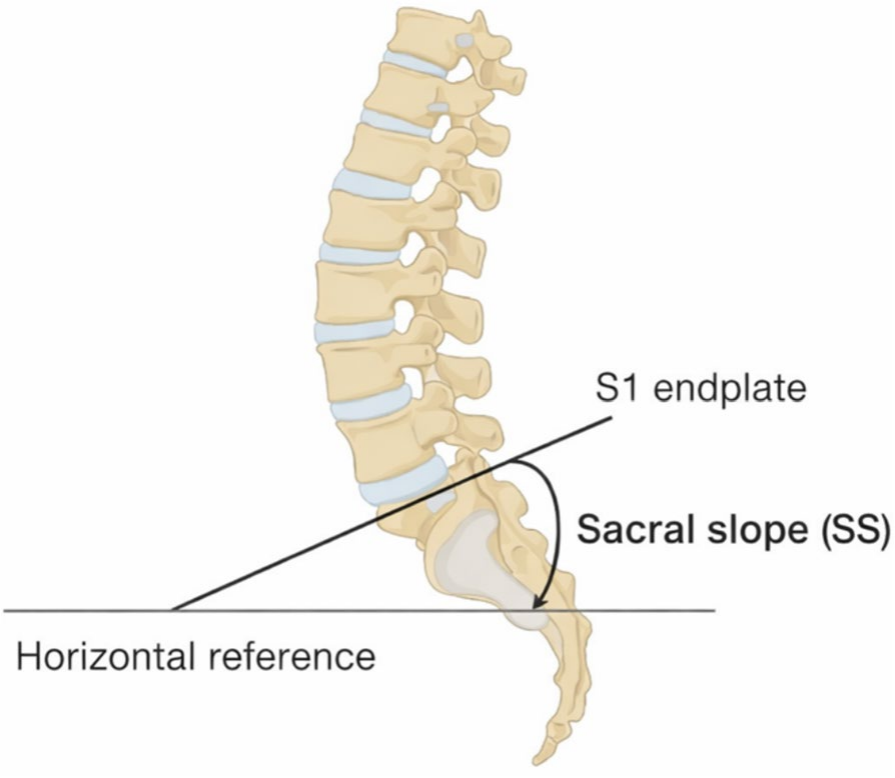
Sacral slope (S1: first sacral vertebra; SS: sacral slope)

Functional imaging does not alter the hierarchy of sagittal alignment drivers but rather shows the adaptive capacity of the spinopelvic unit [57]. Standing-sitting pelvic radiographs and lumbar flexion–extension views demonstrate how pelvic orientation changes with spinal mobility or stiffness, confirming that pelvic tilt is an expression of sagittal balance strategies rather than an independent primary driver [58, 59]. Modern imaging, especially EOS technology, allows for simultaneous assessment of the spine, pelvis, and limbs under load [60, 61]. Parameters such as sacral slope, pelvic incidence, and their mismatch with lumbar lordosis reveal sagittal imbalance that might otherwise be hidden [62]. Sacral slope is defined as the angle between the superior endplate of the first sacral vertebra (S1) and the horizontal reference line on a weight-bearing lateral radiograph (Fig. 4).

Robotic and navigated TKA improve accuracy but still rely on local landmarks up to, but not including, the centre of the femoral head; without considering the more proximal pelvic behaviour, they might risk functionally inappropriate alignment [26, 63]. The ongoing shift towards kinematic alignment and patient-specific instrumentation in TKA has created a need for a broader understanding of how pelvic posture and movement influence lower limb mechanics [64]. Pelvic parameters should be considered alongside hip–spine–knee alignment to optimise component orientation and ligament balancing [9, 10, 64]

### Spinopelvic mobility and functional outcomes

Spinopelvic mobility remains a critically underappreciated factor in TKA, despite its long-standing recognition in hip and spine surgery [65, 66]. The ability of the pelvis to rotate and adapt relative to the spine and femur is crucial for maintaining sagittal balance during everyday activities, such as sitting, standing, and walking [54]. It is a key determinant of sagittal balance, load distribution, and joint mechanics across the lumbosacral junction and hips. This dynamic interplay between lumbar curvature, sacral slope, and pelvic orientation enables the body to modulate the mechanical axis of the lower limb without overloading the knee [67]. When spinopelvic motion is preserved, posterior pelvic rotation during flexion-based tasks helps maintain the CoM over the base of support, with minimal compensatory stress transferred to the knee. However, when this mobility is lost, whether from spondiloarthritis, previous spondylodesis, or inflammatory spondyloarthropathy, the knee becomes a secondary compensator for the stiffened proximal chain [68]. Patients with lumbar kyphosis and posterior pelvic tilt typically exhibit persistent knee flexion to maintain upright posture and visual horizon. The order of compensation (hip extension, then knee flexion, then ankle dorsiflexion) can vary between individuals [69]. In some cases, knee flexion may occur even before hip extension is fully exhausted [68]. These flexion patterns are frequently misinterpreted as stable joint contractures, leading to inappropriate surgical correction through distal femoral resection or posterior capsular release, which may result in gap imbalance or residual flexion postoperatively [70]. Conversely, patients with exaggerated anterior pelvic tilt and lumbar hyperlordosis often display knee hyperextension at rest, with overstretched posterior structures and reduced flexion reserves [15]. Intraoperatively, these features may falsely suggest excessive extension gaps, prompting unnecessary tibial slope reduction or excessive posterior constraint, ultimately destabilising the joint during flexion.

**Fig. 5:**
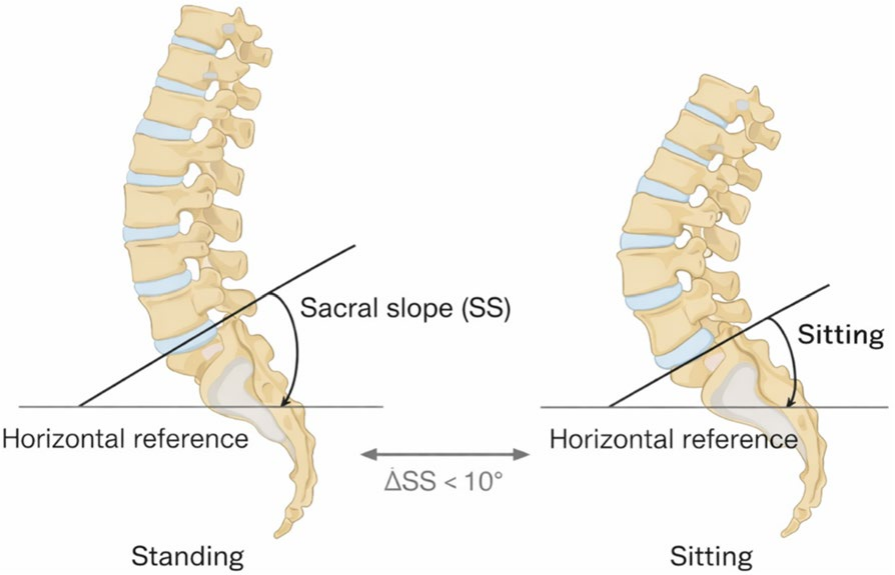
Sacral slope (SS) in standing and sitting positions. The change in SS (ΔSS) between standing and sitting reflects spinopelvic mobility; a ΔSS < 10° indicates reduced sagittal adaptability and functional spinopelvic stiffness

The biomechanical effects of diminished spinopelvic mobility impact gait, stair climbing, and standing up from a chair, with noticeable decreases in stride length, uneven loading, and compensatory trunk movements [71]. These functional limitations, although sometimes subtle, can considerably reduce patient satisfaction, especially when the implanted knee arthroplasty functions well but does not align seamlessly with the patient’s overall posture. Accurate identification requires a combination of clinical examination and imaging. A change in sacral slope between standing and sitting of less than 10 degrees reliably indicates stiffness, although functional impairment may also result from limited hip extension or hamstring contracture (Fig. 5) [66].

To assist in clinical interpretation of spinopelvic dynamics, the classification system proposed by the American Academy of Orthopaedic Surgeons categorises patients based on sagittal balance and pelvic mobility into distinct types (Type IA, IB, IIA, and IIB) [72, 73]. This framework distinguishes between balanced and imbalanced spinal alignment and between mobile and stiff spinopelvic units, offering a practical approach to evaluating compensatory capacity [72, 73]. Of particular interest to total knee arthroplasty is Type IIB spinopelvic imbalance, characterised by sagittal misalignment and reduced pelvic mobility, often linked with flat-back deformity and a fixed posterior pelvic tilt. In this scenario, compensatory mechanisms at the hip and pelvis are exhausted, transferring the adaptive challenge distally to the knee [72, 73].

In practical terms, this classification can guide preoperative workup. Standing and sitting lateral radiographs are advisable in patients with prior lumbar fusion, ankylosing spondylitis, persistent low back pain, fixed flexed posture, or clinically suspected knee–hip–spine syndrome [72, 73]. A ΔSS < 10° between standing and sitting indicates functional spinopelvic stiffness and should prompt consideration of modified alignment targets, such as a slightly flexed femoral component, preservation of posterior condylar offset, and cautious adjustment of tibial slope, to accommodate the patient’s fixed sagittal pattern [26, 72–74]. Patients classified as Type IIB warrant particular attention, as their reduced compensatory reserve increases the risk of postoperative dissatisfaction if strict mechanical alignment is pursued [72, 73, 75].

Imaging technologies, such as EOS or full-body low-dose biplanar radiographs, allow simultaneous visualisation of the entire kinematic chain under load, helping identify mismatches between the lower limb and the proximal trunk [60, 76]. For surgical planning, adjustments may include maintaining slight femoral flexion, preserving the posterior condylar offset, or moderating the tibial slope according to the patient’s posture rather than classical textbook geometry [74].

Postoperative rehabilitation must address the underlying cause, using protocols that include spine mobilisation, hip flexibility, and proprioceptive retraining [77, 78]. The standard protocol, which focuses solely on the knee, may be insufficient to restore functional extension or gait symmetry. Increasing clinical evidence indicates that patients with unrecognised spinopelvic stiffness report poorer outcomes and dissatisfaction, not because of technical errors, but because compensatory knee postures are often mistaken for primary deformities [75]. In such cases, efforts to restore normal ranges of motion may conflict with spinopelvic stiffness or sagittal imbalance, as the spine and pelvis cannot be corrected through the knee alone [39, 70]. A comprehensive assessment of spinopelvic behaviour, before, during, and after surgery, is therefore essential to ensure biomechanical harmony and long-term success in knee arthroplasty.

### Coronal pelvic asymmetries and malalignment

**Fig. 6:**
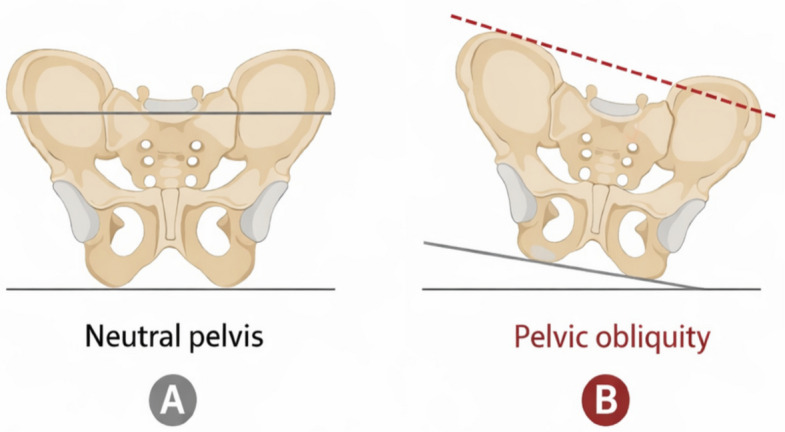
Pelvic obliquity. **A** Neutral pelvis with symmetric iliac crest height. **B** Coronal pelvic obliquity is illustrated by an inclined intercristal line, resulting in asymmetric hip height

**Fig. 7:**
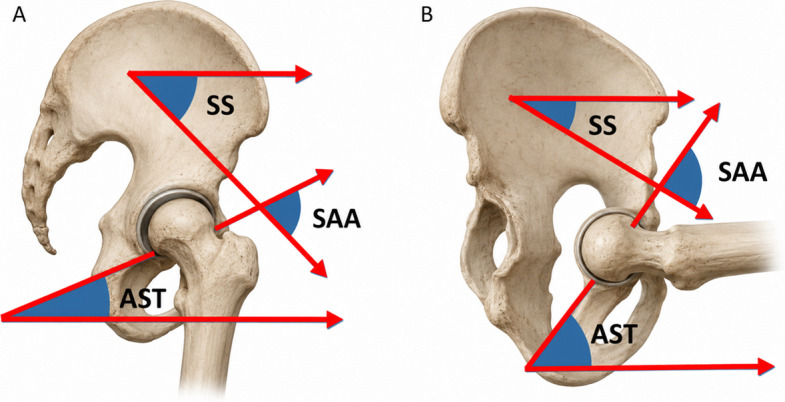
Sagittal spine-pelvis-hip orientation during **A** standing (sacral slope (SS), sacro-acetabular angle (SAA), and acetabular sagittal tilt (AST); **B** Changes in SS, SAA, and AST with the sitting position

**Fig. 8:**
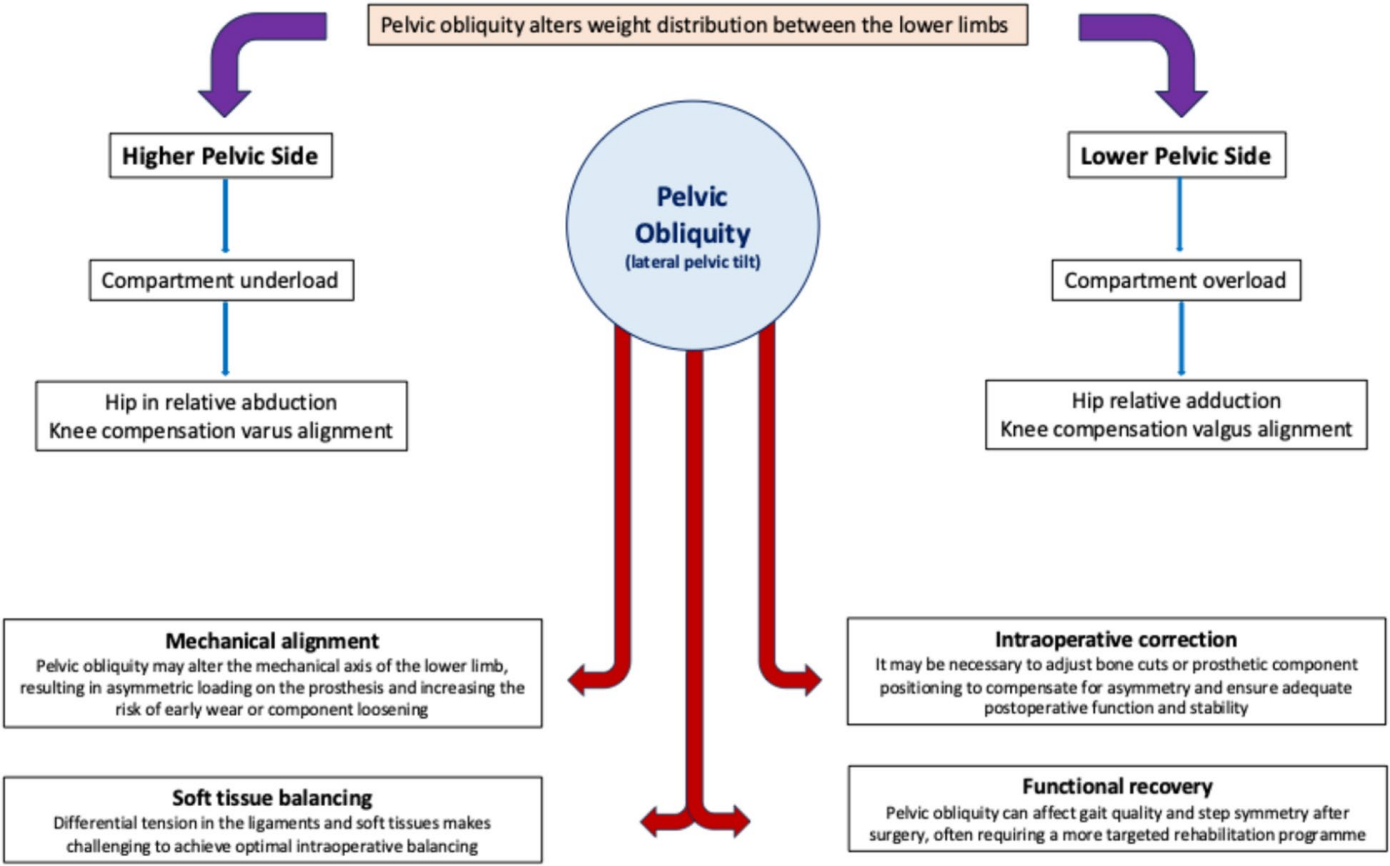
Effects of pelvic obliquity on limb length perception, coronal knee alignment, and asymmetric loading

In pelvic coronal asymmetries, one side of the pelvis is positioned higher than the other in the coronal plane [79, 80]. Pelvic asymmetry on the lateral side, also known as lateral pelvic tilt or, more commonly, pelvic obliquity (Fig. 6), is often underrecognized in TKA, although it may significantly affect limb alignment and influence soft-tissue balance and implant positioning [11]. Pelvic obliquity can result from structural causes, such as leg length discrepancy, scoliosis, or congenital pelvic deformities, which produce a fixed asymmetry in bony alignment [81]. Alternatively, functional causes arise from muscle imbalances, poor posture, joint restrictions, or compensatory mechanisms related to pain or injury, and are typically reversible with appropriate therapy [82]. Pelvic obliquity may affect spinal alignment, gait mechanics, and lower limb loading, potentially contributing to musculoskeletal disorders [80, 83]. A dropped hemipelvis necessitates functional shortening of the ipsilateral limb to maintain a level stance. This adaptation may occur through coronal-plane adjustments at the knee, the direction of which depends on the patient’s native alignment phenotype, with varus or valgus deviation serving as alternative mechanisms to modify effective limb length [84]. Conversely, a dropped hemipelvis results in functional shortening, often associated with varus deformity or hyperextension of the ipsilateral knee (Fig. 6). These compensatory mechanisms, while dynamic in origin, may become structurally ingrained over time and may also interact with sagittal spine–pelvis–hip orientation, including sacral slope, sacro-acetabular angle, and acetabular sagittal tilt during postural transitions (Fig. 7). If not recognised during preoperative assessment, they may lead to misinterpretation of coronal deformities as primary joint pathologies, prompting unnecessary or asymmetrical bone resections. The resulting biomechanical implications for coronal knee alignment and load distribution are illustrated in Fig. 8.

Axial rotation of the pelvis adds another layer of complexity, as it alters the orientation of the acetabulum and femoral version, disrupting standard rotational landmarks such as the posterior condyles or the transepicondylar axis [35]. In TKA workflows that rely mainly on fixed bony references, failure to account for pelvic rotation may lead to femoral malrotation and downstream complications, such as patellar maltracking or flexion imbalance [85]. In contrast, gap-balanced or functionally positioned techniques may partly mitigate, but not eliminate, these effects [86, 87].

Radiographic evaluation must extend beyond the knee. Full-length standing radiographs may reveal iliac crest asymmetry, sacral base tilt, or lateral deviation of the pubic symphysis, all of which suggest coronal pelvic imbalance [88, 89]. In more complex cases, EOS imaging or functional gait analysis can help clarify whether the deformity is structural or compensatory, thereby distinguishing between fixed contractures and posture-driven misalignments [61, 90]. In monocompartmental knee arthroplasty, correction to neutral alignment without considering an oblique pelvis may create a perceived leg length discrepancy or even cause contralateral overload [91]. In bicompartmental knee arthroplasty, asymmetrical correction may exacerbate postural imbalances or induce spinal compensation, particularly in the presence of degenerative scoliosis or prior spinal instrumentation [92, 93]. Surgical strategy should be adapted accordingly, embracing a functional alignment philosophy that, in the coronal plane, respects the patient’s habitual posture rather than imposing mechanical symmetry at all costs, and, in the sagittal plane, acknowledges the hierarchy of compensatory mechanisms without perpetuating fixed contractures. Interpretation of coronal limb alignment must also consider constitutional knee geometry. A wide range of varus and valgus configurations fall within physiological limits and should not be automatically regarded as pathological [94]. The concept of Coronal Plane Alignment of the Knee (CPAK) has highlighted how native alignment phenotypes influence both load distribution and compensatory capacity [95, 96]. Accordingly, coronal adaptations to pelvic obliquity or functional limb length demands are alignment-dependent, as identical biomechanical requirements may be accommodated through different joint-level strategies in varus- or valgus-predominant phenotypes [97]. Recent three-dimensional CT-based phenotyping studies have further demonstrated that distinct arthritic patterns are closely associated with pre-existing knee morphologies, underscoring that coronal deformity in osteoarthritis often represents phenotype-specific progression rather than a uniform pathological process [97, 98]. Postoperative rehabilitation must also address these asymmetries through targeted neuromuscular training, core strengthening, and proprioceptive retraining to avoid the re-establishment of maladaptive gait patterns. Persistent pain, asymmetric loading, or subjective dissatisfaction after TKA often reflect uncorrected or newly unmasked coronal pelvic issues, which, if left untreated, can undermine the mechanical success of the arthroplasty procedure.

### Clinical relevance and surgical implications

Recognising pelvic biomechanics in TKA is not merely a theoretical concern but a crucial factor in achieving consistent and durable outcomes. The pelvis influences limb alignment in all planes and dynamically interacts with the spine and hips [99, 100]. When pelvic posture is off, from tilt, rotation, or stiffness, relying solely on bony landmarks of the knee for alignment can be misleading. Traditional mechanical alignment, though useful in many cases, might cause imbalance in patients with spinopelvic rigidity or coronal asymmetry [75]. These patients may appear malaligned on radiographs when examined in isolation, yet they function well in their usual posture. Applying textbook correction may disrupt this balance, leading to soft-tissue imbalance, extensor mechanism dysfunction, or unnatural joint biomechanics. A functional alignment strategy, based on the patient’s overall kinematics rather than mechanical standards, may allow more precise adjustment of component position and slope [26, 101]. For those with fixed posterior tilt and chronic knee flexion, it might be wise to accept a slightly flexed position and avoid overly aggressive distal femoral cuts [102]. Conversely, patients with hyperlordosis and anterior tilt may require adjustments, such as modifying the tibial slope or enhancing the insert constraint, to prevent posterior instability [75]. Additionally, femoral component rotation should be tailored to each patient, particularly when pelvic rotation or abnormal acetabular alignment is present, as standard landmarks may no longer be relevant [103, 104]. A concise summary linking common pelvic biomechanical patterns to their typical knee manifestations and corresponding intraoperative considerations is provided in Table 2.

**Table 2:** Pelvic biomechanics, knee manifestations, and surgical implications in TKA

Biomechanical pattern	Typical knee manifestation	Surgical implication/recommendation
Posterior pelvic tilt	Apparent knee hyperextension or fixed flexion posture	Avoid excessive distal femoral resection; accept mild residual flexion if functionally compensated
Anterior pelvic tilt/hyperlordosis	Tight extension gap; altered extension mechanics	Modify tibial slope cautiously; avoid over-tightening the extensor mechanism; consider increased insert stability
Spinopelvic stiffness with sagittal imbalance	Knee acts as a secondary compensator; difficult gap balance	Avoid strict mechanical alignment; favour functional alignment, respecting habitual posture
Coronal pelvic obliquity	Functional limb length discrepancy; asymmetric coronal loading	Avoid symmetric correction to neutral; respect coronal asymmetry
Pelvic axial rotation	Unreliable femoral rotational landmarks	Individualise femoral component rotation; do not rely solely on standard references

A key intraoperative consideration is the distinction between fixed structural deformity and dynamic functional compensation, as failure to recognise this difference may lead to overcorrection [81, 105]. In the coronal plane, pelvic obliquity that resolves on supine examination or with leg-length equalisation is likely functional and should not prompt asymmetric bone resection, whereas a fixed obliquity from scoliosis or true limb-length discrepancy may justify accepting non-neutral alignment to preserve overall postural balance [20, 106]. In the sagittal plane, persistent knee flexion accompanied by a reducible hip flexion contracture or correctable lumbar kyphosis usually reflects compensation and should not be addressed by additional distal femoral resection, which risks joint-line elevation and gap imbalance [107, 108]. Examination under anaesthesia together with full-length weight-bearing imaging in both standing and sitting positions helps clarify whether the deformity is reducible, and the surgical strategy should be adapted accordingly [44, 109].

Gap balancing, particularly in navigated or robotic surgery, should be approached with contextual awareness. Asymmetric tension does not always indicate ligament pathology, and soft tissue resistance may result from pelvic-driven positional variations [83]. Understanding the source of perceived imbalance helps prevent overcorrection and avoid unnecessary constraint. Implant selection should also consider the neuromuscular demands imposed by altered pelvic mechanics. In patients with limited compensation capacity, such as those with spondylodesis, more constrained designs may provide improved functional stability. Preoperative planning should always include assessment of sacral slope, pelvic incidence, and lumbar lordosis, ideally with full-length imaging, to anticipate compensatory patterns and plan accordingly [110, 111]. Postoperative protocols should be tailored to the biomechanical profile. Patients with sagittal imbalance may need retraining of lumbar mobility and gluteal strength, while those with coronal asymmetry often benefit from targeted gait correction and trunk stabilisation. Ultimately, recognising the pelvis as a dynamic and influential structure enables surgeons to move beyond two-dimensional alignment goals, fostering a more comprehensive and physiologically attuned arthroplasty philosophy. In this context, long-term function and patient satisfaction are more likely to reflect the true success of the intervention.

### Limitations of the current evidence and future prospects

The current understanding of pelvic biomechanics in TKA remains limited. Research on pelvic tilt, spinopelvic mobility, and coronal asymmetry remains sparse, fragmented, and often methodologically inconsistent. Most studies rely on small samples and retrospective designs, using static, two-dimensional imaging to investigate a system that is inherently dynamic and three-dimensional. Moreover, there is currently no consensus regarding optimal measurement protocols, with studies variably using EOS imaging, conventional long-leg radiographs, computed tomography (CT)-based three-dimensional evaluation, or functional imaging approaches. This heterogeneity limits comparability across studies and complicates the definition of clinically meaningful thresholds for pelvic tilt, spinopelvic mobility, and coronal asymmetry. Sagittal tilt is frequently assessed in isolation, without considering the interaction between lumbar mobility, sacral slope variation, and hip extension capacity, all of which influence knee mechanics during daily activities. Spinopelvic stiffness is often inferred from a single radiograph rather than evaluated through dynamic, motion-sensitive tools. Emerging technologies may help address these limitations. AI-based motion analysis systems, including modern markerless computer vision approaches, enable high-temporal-resolution dynamic measurement of pelvic rotation, trunk inclination, and knee loading patterns during daily activities.

Early validation studies show that such systems can detect subtle deviations in sagittal and coronal mechanics, such as early pelvic rotation during stance or asymmetric knee loading, which are not visible on static radiographs [112, 113]. Wearable inertial measurement units (IMUs) likewise allow continuous monitoring of pelvic tilt variation, gait asymmetry, and cumulative joint loading over extended periods, capturing real-world compensatory patterns indicating fatigue or reduced spinopelvic adaptability [114, 115]. Intraoperatively, robotic-assisted systems already record high-fidelity, real-time tibiofemoral kinematics, and future research should explore how these kinematic signatures interact with pre- and postoperative spinopelvic behaviour. Integrating these modalities could bridge the gap between static imaging and dynamic function, enabling more accurate identification of compensatory chains and supporting personalised alignment strategies.

Similarly, coronal pelvic asymmetries and axial rotation, caused by scoliosis, sacroiliac dysfunction, or soft-tissue imbalance, are seldom quantified despite their evident potential to affect limb alignment and prosthetic load. There is little consensus on measurement methods, thresholds for clinical relevance, or how they should influence implant selection, positioning, or constraint choice. Few studies focus on alignment or component orientation and lack long-term patient follow-up, leaving unanswered questions about their impact on functional recovery, wear patterns, and revision risk. Moreover, patient-specific factors such as sex, age, spinal morphology, neuromuscular adaptability, and compensatory mechanisms are rarely considered, even though they are likely to affect the biomechanical influence of pelvic parameters. The pelvis is often viewed as a static structure, but in reality, it continuously adapts to structural deformities and functional demands. Trunk inclination, hip stiffness, leg dominance, and foot posture all influence how pelvic orientation affects knee kinematics. Yet, these factors are rarely incorporated into preoperative planning or postoperative care. Rehabilitation strategies continue to focus solely on the knee, overlooking upstream contributors to imbalance or fatigue involving the spine, pelvis, and hip, and neglecting distal adaptations at the ankle and subtalar joints, where calcaneal alignment plays a vital role in accommodating sagittal and coronal demands during gait. Ultimately, there is a disconnect between the complex biomechanics of the kinetic chain and the oversimplification of current clinical protocols.

To progress, research must adopt a broader and more integrated perspective. Prospective, multicentre studies are necessary to evaluate the influence of pelvic tilt, spinopelvic stiffness, and coronal asymmetry using standardised protocols that combine standing and seated imaging with dynamic gait analysis. Such studies should be sufficiently powered and stratified by relevant patient variables, following participants over several years to assess functional outcomes, implant performance, and patient satisfaction. Clinical research should also examine how different alignment philosophies, mechanical, anatomical, or functional, perform across various pelvic profiles. Experimental work, including modelling studies and intraoperative navigation data, could help clarify how pelvic orientation impacts soft-tissue balance or joint loading. Concurrent efforts in rehabilitation science should focus on core stability, lumbar mobility, and pelvic control, investigating how these factors might be optimised to improve post-operative gait and reduce compensatory stress. Practical tools for routine use, such as simplified posture assessments or wearable sensors, would encourage wider clinical adoption. Finally, stronger collaboration between spine, hip, knee, and ankle specialists is essential to define shared guidelines, decision thresholds, and integrated care pathways. Only through such an interdisciplinary, evidence-based approach can complex biomechanical insights be translated into tangible clinical benefits for patients undergoing knee replacement.

## Conclusion

Pelvic biomechanics has historically received limited attention in TKA planning, despite its potential influence on alignment, balance, and function. However, the pelvis is not passive: it controls posture, compensates for deformity, and transmits forces that ultimately shape the knee’s mechanical environment. Ignoring sagittal tilt, coronal asymmetries, or spinopelvic stiffness risks reducing arthroplasty to a static and compartmentalised procedure, unaware of the chain of interdependence that underpins human movement. As surgical philosophy shifts from mechanical neutrality to functional optimisation, incorporating pelvic alignment, mobility, and compensatory mechanisms into preoperative assessments can significantly improve both short- and long-term outcomes of TKA. Robust clinical studies are necessary to determine how pelvic parameters affect prosthetic performance and develop evidence-based strategies that account for the entire kinetic chain.

## Data Availability

All relevant data are included in the article.
